# Beyond performance: Emotions before and after semi‐high‐stakes mathematics testing among school‐aged students

**DOI:** 10.1111/bjep.70043

**Published:** 2025-10-29

**Authors:** Reetta Kyynäräinen, Santeri Holopainen, Jari Metsämuuronen, Umar Bin Qushem, Mikko‐Jussi Laakso, Katarina Alanko

**Affiliations:** ^1^ Turku Research Institute for Learning Analytics University of Turku Turku Finland; ^2^ Department of Chemistry University of Turku Turku Finland

**Keywords:** assessment, competence belief, emotions, gender differences, semi‐high‐stakes testing, test performance

## Abstract

**Background:**

Previous research has shown that testing differs significantly from other classroom activities and is associated with heightened negative emotions and lower levels of positive emotions. However, relatively little is known about students' emotions surrounding testing, particularly in higher‐stakes assessment settings.

**Aims:**

This study aims to examine how students' levels of four emotions (i.e., happiness, relaxation, anxiety and boredom) develop from pre to‐post‐test, and it investigates how individual factors (i.e., gender, grade level, perceived mathematical competence and test performance), impact students' emotional states and moderate their emotional trajectories.

**Sample:**

The sample (*N* = 2179) consists of 692 third‐grade, 605 sixth‐grade, 413 eighth‐grade and 469 ninth‐grade students from various schools across Finland, who participated in a digital, semi‐high‐stakes, end‐of‐year mathematics assessment.

**Methods:**

An in‐situ approach was used to assess students' emotions immediately before and after testing. Analyses were conducted using linear mixed‐effects modelling to account for the repeated‐measurements structure.

**Results and Conclusions:**

Students generally reported lower positive emotions after the assessment. The measured individual factors significantly predict both students' emotional states and their development during the assessment. Boys reported higher levels of positive emotions and lower anxiety, while younger students remained more positive during the assessment. Students who perceived themselves as competent experienced higher levels of positive and lower levels of negative emotions, whereas students who performed poorly showed a decline in positive emotions during the assessment. Future research could focus on whether support for emotional regulation affects student performance in test situations.

## INTRODUCTION

Emotions actively influence cognitive processes like attention and memory, as well as affective processes such as motivation, particularly in evaluative settings (Pekrun, 2006; Pekrun et al., 2002). There is an abundance of previous research on students' emotions—particularly anxiety—related to assessment and testing in mathematics (Halme et al., 2022; Putwain, 2008; Putwain et al., 2021; Schutz & Davis, 2000; Vogl & Pekrun, 2016). However, there is still limited research (see Goetz et al., 2007; Kleine et al., 2005) on emotional trajectories surrounding testing situations, especially concerning the development of emotions and the factors contributing to these shifts (Schmid et al., 2025; Vogl & Pekrun, 2016). Moreover, previous research has predominantly focused on negative emotions, overlooking positive affect, especially following assessment (Pekrun et al., 2002; Vogl & Pekrun, 2016). Understanding these emotional trajectories is essential, as they may reflect shifts not only in students' performance but also in broader affective constructs such as engagement or persistence in mathematics.

This study explores how elementary and middle school students' emotions shift before and after a large‐scale, semi‐high‐stakes math assessment. The assessment provides teachers with a final grade suggestion for each student based on their performance on the test. Furthermore, we focus on the roles of gender, grade, test scores and perceived math competence. By examining emotional changes from pre‐ to post‐assessment, the research aims to understand why some students remain calm or excel under pressure, helping identify those who may need targeted emotional support to promote both academic and emotional development.

### Emotions

Emotions fall under the affective learning domain, which also contains concepts like moods, beliefs, attitudes, motivation and interest. An emotion, in contrast to a mood, is of a shorter duration, more fluid nature and has a more specific focus. Typically, emotions are understood as transient states triggered by a specific stimulus, but they can also reflect individuals' dispositions to experience such states in comparable contexts. These momentarily, transient states are referred to as state emotions, while the latter constitutes what is known as trait emotions (Pekrun et al., 2018).

In addition to stability, emotions are often categorized based on valence and arousal (Pekrun et al., 2018). Valence refers to the intrinsic positivity or negativity of an emotional experience, that is, how pleasant or unpleasant the emotion feels, and it can be used to categorize emotions as positive or negative. Arousal, on the other hand, distinguishes emotions based on shifts in one's physiological activity when experiencing the emotion. Activating emotions prompt quick reactions, while deactivating emotions promote rest (Ketonen et al., 2023). These two dimensions can be used to separate emotions into four categories in a two‐dimensional emotional map: positive activating (such as happy, excited), positive deactivating (e.g., relaxed, relieved), negative activating (e.g., anxious, frustrated) and negative deactivating (e.g., bored, hopeless) (Pekrun et al., 2018). We chose to examine four emotions—happy, relaxed, anxious and bored—as representatives of the four quadrants of this two‐dimensional circumplex model.

Third, Pekrun et al. (2018) identify object focus, the trigger or antecedent of the emotion, as a key factor in addressing emotions at school. In instructional settings, emotions are triggered by the ongoing academic tasks and can be defined as academic emotions. Emotions related to assessment, however, may be classified as achievement emotions, tied to students' expectations and experiences of success or failure (Pekrun, 2006). Nonetheless, emotions are holistic by nature, and without further information from the learner, object‐based distinctions should be made with caution.

Emotions arise in response to external triggers, but internal factors can impact individuals' emotional regulation, too (Harley et al., 2019). According to previous research, students' achievement, self‐perceptions, age and gender, for instance, can predict their emotional states or emotional responses to certain triggers. In mathematics, high‐performing students are less anxious (Erturan & Jansen, 2015; Pekrun et al., 2018), students who feel competent experience more positive emotions (Pekrun et al., 2018; Vogl & Pekrun, 2016), girls experience particularly more trait‐like test‐anxiety (Erturan & Jansen, 2015; Frenzel et al., 2007; Goetz et al., 2013), and younger students express higher levels of positive emotions and lower levels of negative emotions (Mata et al., 2022). However, prior studies suggest that these individual characteristics might not impact dynamic state emotions as significantly as they impact trait‐emotions (see Goetz et al., 2013; Tulis & Ainley, 2011). This could be due to the fact that some individual factors can contribute to stereotypes which influence how likely students are to overestimate, for example, their habitual anxiety (cf. Goetz et al., 2013). In addition, in contrast to more stable trait emotions, adolescents' situational state emotions show substantial within‐person variability across settings and companions. Situational factors and the proximal environment account for large fluctuations in state affects, repeatedly demonstrated by, for example, experience‐sampling studies (Dirk & Nett, 2022; Mölsä et al., 2025; Vilhunen et al., 2022).

### Emotions during learning and assessment

Although primarily affective by nature, emotions also encompass cognitive, physiological, motivational and behavioural components (Brun et al., 2008; Pekrun et al., 2017). As such, they potentially influence students' thinking, motivation and actions, promoting or hindering learning and test performance (Jarrell & Lajoie, 2017; Pekrun et al., 2002). They shape key cognitive processes such as concentration, attention, memory and decision‐making (Pierson et al., 2023; Shuman & Scherer, 2013; Vilhunen et al., 2023; Vogl & Pekrun, 2016).

Generally, positive emotions relate positively to fundamental metacognitive strategies, such as elaboration and critical thinking (Pekrun, 2006; Pekrun et al., 2002), which are vital in a test setting. The connection between experiencing negative emotions and learning is not as consistent as, for instance, negative activating emotions (e.g., anxiety) can foster efficient metacognitive strategies (Pekrun et al., 2002; Tulis & Fulmer, 2013), whereas negative deactivating emotions (e.g., boredom) can be detrimental (D'Mello & Graesser, 2011; Ketonen et al., 2023; Pekrun et al., 2010). Learning could be promoted by maintaining negative emotions at adequate levels. However, students might need additional pedagogical scaffolding to regulate the levels and duration of negative emotions—support that is rarely available during assessment (D'Mello et al., 2014).

Pekrun's control‐value theory of achievement emotions (Pekrun, 2006) has been widely used as a theoretical lens for studying affects related to assessment in mathematics, because it identifies relationships between motivational factors, emotions and learning outcomes in students. Students' achievement emotions—enjoyment, boredom and anxiety—in mathematics are predicted by intrinsic values (e.g., interest) and sense of control, which are in turn linked to their achievement outcomes in testing (Putwain et al., 2021). They are also connected to students' self‐concept, including students' perceptions of their competence in mathematics, which is positively associated with mathematics achievement (Frenzel et al., 2007; Van Der Beek et al., 2017; Zhang et al., 2023). Thus, experiencing academic success or failure in an assessment could trigger reappraisal of one's competence, shaping students' self‐concept and emotions (cf. Frenzel et al., 2007; Pekrun et al., 2018; Putwain et al., 2021). In general, mathematics self‐concept has been found to weaken through the years of elementary and secondary education, accompanied by the increase of trait‐like math anxiety (Kaur et al., 2022; Nagy et al., 2010).

Previous research has shown that testing situations differ significantly from other types of classroom activities (Beymer et al., 2021; Schutz & Davis, 2000; Vogl & Pekrun, 2016). Generally, students associate high importance with tests, quizzes and their outcomes (Beymer et al., 2021; Schutz & Davis, 2000). Test settings might promote a lower sense of control over the outcome, perhaps because students have limited opportunities to choose how to behave or what to direct their attention to (Beymer et al., 2021). Additionally, students associate heightened negative emotions and lower levels of positive emotions with assessment (Beymer et al., 2021; Schutz & Davis, 2000). However, much of this knowledge is cross‐sectional, with limited research exploring how emotions evolve across the testing situation.

Nevertheless, a few studies have previously investigated students' emotions before, during and after testing, proposing that students experience heightened levels of negative emotions before testing (see Goetz et al., 2007; Kleine et al., 2005). Goetz et al. (2007) suggest that low math ability predicts higher anxiety during testing and portray a general decline in enjoyment and increases in anger and boredom from pre‐ to post‐testing. Moreover, positive deactivating emotions are typically experienced after achievement tasks (Goetz et al., 2007; Junça‐Silva et al., 2018; Pekrun et al., 2002).

Within this field, it is relevant to distinguish the high‐stakes or low‐stakes nature of the assessment because it can play a key role in predicting students' affective experiences and self‐regulation (Ball, 1995; Putwain, 2008; Schutz & Davis, 2000; Vogl & Pekrun, 2016). If a test significantly affects outcomes like final grades, students may attach greater importance to it, triggering different or more intense emotions. However, much prior research, including studies on emotional development during testing, has been conducted in low‐stakes contexts (see Putwain et al., 2021), which likely lowers student motivation and influences their emotional responses.

Some students can experience trait‐like anxiety or hopelessness in test settings. These emotions can significantly hinder their performance in the test, even risking the validity of mathematics test scores across students with different levels of test anxiety (Vogl & Pekrun, 2016). Previous research states that the intensity of test anxiety could be related to the stakes of the assessment setting, but even more so to how familiar the test setting is to students (Putwain, 2008). Furthermore, trait‐like test anxiety can moderate the levels of students' emotions associated with feeling challenged during a test. According to previous studies, lower test anxiety is associated with more positive emotions and less self‐blame when faced with challenges (Schutz & Davis, 2000).

### Mathematics education and assessment in Finland

In Finland, children typically enter school at the age of seven, after having completed 2 years of pre‐primary education without an academic burden. Unlike in many other countries, children are not typically taught reading, writing, or mathematics in preschool; rather, the academic program begins in school (EDUFI, 2014, 2020, 2022). Accordingly, children enter first grade with a wide range of skills (see Metsämuuronen & Ukkola, 2019, 2022; Ukkola & Metsämuuronen, 2019; Ukkola et al., 2020).

Unlike in most European countries, no high‐stakes, exam‐type evaluation is administered during basic education. Instead, student assessment and evaluation are based on teachers' observations as well as diagnostic and teacher‐made tests (Harju et al., 2025). In general, teachers may use three types of tests: teacher‐made, diagnostic and summative tests. Typically, teachers administer several teacher‐made tests throughout the year before and after math courses. Diagnostic tests, on the other hand, include assessments such as the FUNA‐DB (Functional Numeracy Assessment Dyscalculia Battery; Hellstrand et al., 2024; Räsänen et al., 2021) for numeracy fluency and the MUREA (Multilingual Reading Assessment; Bertram et al., 2025; Salmela et al., 2021) for language fluency. Although not all schools use these tests, tens of thousands of students take the FUNA‐DB alone every year. Third, summative tests are available for some grades from the Finnish Association for Teachers of Mathematics, Physics, Chemistry and Informatics. The DigiEva dataset in this study is one of these summative tests.

The challenge with teacher‐made tests is that they are not comparable across schools and municipalities. The normative National Core Curriculum for Basic Education addresses this issue by providing guidelines for grading (EDUFI, 2020). However, national assessments (Metsämuuronen, 2023) show that ‘assessment cultures’ differ radically between schools and providers. Students with the same level of achievement in mathematics may receive different grades from teachers in different classes, schools and municipalities. The DigiEva assessment aims to tackle this challenge, providing comparable suggestions of the end‐of‐year grade for each student across schools and municipalities.

### The present study

This study aims to investigate how students' emotions develop from pre‐ to post‐assessment in a semi‐high‐stakes mathematics test. Specifically, we focus on four key emotions—happiness, relaxation, anxiety and boredom—and examine how these trajectories are influenced by individual characteristics (gender, grade level, perceived mathematical competence and actual test performance). The study contributes to broadening the range of emotions studied by including an often‐overlooked positive deactivating emotion of relaxation.

The specific research questions are:

1. Is there a significant change in the levels of students' emotions from pre‐ to post‐assessment tasks?
2. Is the change in emotional levels moderated by gender, grade level, competence beliefs and test performance? If so, to what extent?
3. Do gender, grade, competence beliefs and test performance affect students' emotional levels at the general level, regardless of time? If so, to what extent?

The study was guided by the following hypothesis:

Before the assessment, students experience higher levels of activating emotions as they may relate high importance and higher cognitive demands to that situation, compared to post‐assessment, when we expect to see tension‐reducing deactivating emotions.

Individual characteristics and test performance moderate the changes; the effects vary across variables and groups. We further explore how individual characteristics (gender, grade, competence belief, performance) moderate the changes in emotions before and after the test situation.

Individual characteristics predict general level of emotions, so that (a) girls report more anxiousness than boys, (b) younger students report more positive emotions overall than older students, (c) students with low competence beliefs report more negative emotions and (d) better test performance is associated with more positive emotions both before and after testing.

## METHOD

### Participants

The participants were 2179 students from the 3rd (692, 31.8%), 6th (605, 27.8%), 8th (413, 19.0%) and 9th (469, 21.5%) grades from various schools around Finland. The schools were recruited by an open call for participation in the DigiEva pilot, constituting a semi‐representative sample of primary and lower secondary schools in Finland. The ages of the students were typically 9, 12, 14 and 15, respectively. 1052 were boys (48.3%), 1028 were girls (47.2%) and 99 chose not to report their gender (4.5%). 2007 completed the assessments in Finnish (92.1%) and 172 in Swedish (7.9%), depending on the official instructional language in the school. For 1746 participants (80.1%), their home language was the same as the assessments' language, whereas for 158 participants (7.3%), their home language was something different. In addition, 178 came from mixed‐language homes (8.2%; Finnish and Swedish or some other language) and 97 did not report their home language (4.5%). Before analysis, students with missing data on gender or competence belief were excluded (130, 6.0%). Finally, students with missing data on the emotion variables were removed (happy: 236, 11.5%; relaxed: 240, 11.7%; anxious: 249, 12.2%; bored: 246; 12.0%).

The study was conducted following Finnish law and the ethical guidelines of the Finnish National Board on Research Integrity (TENK). Research permission was obtained from the relevant municipalities. Students participated anonymously and voluntarily during regular school hours, and their guardians were informed about the assessments in advance. The principle of informed dissent was upheld, ensuring that students could decline participation without any consequences.

### Data collection

The questionnaire data concerning students' emotional experiences were collected before and after an end‐of‐the‐study‐year mathematics assessment conducted on a digital ViLLE learning platform (see Laakso et al., 2018). This study assesses emotions before and immediately after the test. In the in‐situ questionnaires, students were asked ‘How are you feeling right now…’, instructed to respond concerning the four emotions—happy, relaxed, anxious and bored—on a 5‐point Likert scale with response categories ranging from ‘1 = Not at all’ to ‘5 = Very much’ (cf. Pekrun et al., 2002). In the pre‐assessment questionnaire, students also reported their gender and perceived mathematics competence with one item measure: ‘I am good at mathematics’ (cf. Fennema & Sherman, 1976; Gogol et al., 2014; Metsämuuronen, 2012; Parker et al., 2012), again on a 5‐point scale. This item was originally adopted from the Fennema and Sherman Mathematics Attitudes Scale (1976), and edited for the Finnish context (Metsämuuronen, 2012). This edited item, ‘I think I am good at mathematics’, expressed good predictive validity, having the highest factor loading reflecting students' self‐concept in mathematics (Metsämuuronen, 2012). Thereby, a similar, simplified item was chosen for this study as the single‐item measure of perceived competence in mathematics. At the beginning of the background questionnaire, participants were informed about its items, as well as the items asking about their current emotions during the test. All items were in Finnish or Swedish. The background questionnaire was kept short, with a very limited set of questions (varying in length for different grades; item number varied between 22 and 34). For the emotions, the emotion (in words) appeared on the left‐hand side, and the Likert scale next to it on the right‐hand side. All emotions appeared on the same page.

The test itself forms traditional assessment data, referred to as DigiEva. This was a pilot for a national assessment, which provides teachers with a suggestion of an end‐of‐year final grade for each student. Teachers were encouraged to inform students that their end‐of‐year evaluation would be affected by the math test; however, this was not monitored. The test was composed of multiple sections, each evaluating a different mathematical domain. These six domains represented the core of the Finnish curriculum in mathematics: (1) mental calculations and computational thinking; (2) numbers and numerical operations; (3) algebra; (4) functions (not included in the third and sixth grade tests); (5) geometry and measurement and (6) statistics and probability. From this perspective, the construct validity of the tests is ensured. The test was administered to students in grades 3, 6, 8 and 9 in April 2025. The entire DigiEva battery of tests, including background questions and 15‐minute research‐oriented test batteries, took one 45‐minute lesson for third and sixth graders and two 75–90‐min lessons for eighth and ninth graders. During the pilot phase, the time on task was studied, and the number of items was determined so that all students would have enough time to answer all questions. An additional 15 min (the lesson break) was allotted for students who needed more time to finish the test. Each grade had three linked test versions, and the scores were equated so that the average 9th grader received a standard score of 0. Experienced mathematics teachers selected the tasks from the item banks so that the content would be relevant and emphasize application‐type tasks over memorization. The wording of the test items was controlled to avoid ambiguity, excess and complexity. This was done with students with low language skills and reading ability in lower grades in mind.

Finland's national evaluation and assessment system related to learning outcomes is based on national representative samples and low‐stakes tests (Metsämuuronen, 2009; Metsämuuronen & Ukkola, 2019). The low‐stakes characteristic stems from the fact that test results do not affect student selection processes or prospects. Conversely, the DigiEva study in 2025 is partly low‐stakes testing and partly semi‐high‐stakes testing. The low‐stakes characteristic stems from the same fact as in the national assessment: the test score itself is not used in any selection process. However, DigiEva's semi‐high‐stakes characteristic stems from the fact that teachers were encouraged to use the test results when finalizing the end‐of‐year summative evaluation of students. This was suggested to motivate students to perform their best. To this end, teachers were provided with a grading tool that suggested a mark (on a scale of 5–10) for students based on their performance on the test. This suggested grade was based on grade‐wise quantiles of the score distributions and the corresponding quantiles of the teachers' grades in the national register. For example, 13.3% of the best third‐grade students received a mark of 10 (‘excellent’), while 11.0% of the best ninth‐grade students received a mark of 10.

### Analytical approach

To assess the representativeness of the final samples included in the analysis, we compared excluded and included participants in terms of grade, gender, mathematics competence belief, and test score for each emotion separately, as the amount of missing data varied between emotion variables. Pearson's chi‐squared tests of independence were performed for the categorical variables (grade, gender and competence belief), and analyses of variance were performed for the test score.

To account for the repeated measurements structure in the data (two responses nested within students), we employed linear mixed‐effects modelling (LMM) with a random intercept for participants utilizing lme4 (Bates et al., 2015) and lmerTest (Kuznetsova et al., 2017) packages in R 4.5.0 software (R Core Team, 2023). We fitted separate models for each emotion. The emotion levels (on Likert scale) were the dependent variables, and time (‘before’ or ‘after’ the assessment), grade, gender, standardized test score (*M* = 0, SD = 1), competence belief in mathematics, and their possible interactions were used as predictors. We treated time, grade, gender and competence belief as categorical variables and test score as a continuous variable. In the full models (Tables A1, A2, A3, A4), the categorical variables were effect coded, meaning that each level of a variable was compared to the grand mean of that variable. To draw meaningful interpretations, emotion levels were treated as continuous variables, although this violates the normality assumption for LMMs. However, especially since our sample was large, we referred to studies suggesting that parametric statistics can be safely used even when assumptions are violated (Norman, 2010; Sullivan & Artino, 2013). The appropriateness of an LMM approach was supported by the estimated variance attributable to individual participants and the intraclass correlation coefficients for each emotion: .50 (happiness), .47 (relaxation), .51 (anxiety) and .41 (boredom), indicating substantial within‐person clustering. Table 1 contains the descriptive statistics and correlations for the emotion variables.

**TABLE 1:** Descriptive statistics and correlations for the emotion variables.

Variable	*N*	*M*	SD	1	2	3	4
1. Happy	3626	2.78	1.33	–			
2. Relaxed	3618	2.67	1.32	.64	–		
3. Anxious	3600	2.01	1.25	−.22	−.24	–	
4. Bored	3606	3.01	1.45	−.39	−.27	.23	–

*Note*: All correlations were statistically significant at the .001 level.

Abbreviations: *M*, mean; *N*, sample size; SD, standard deviation.

We utilized a forward‐elimination type of approach for selecting the final model. For each emotion, we started with a model with only the main effects of all predictors and the interactions between time and the other predictors, because we were exclusively interested in how the other predictors moderated the effect of time. Then, we iteratively added three‐way interactions that always included the time variable one at a time to the model. If a three‐way interaction was not significant, but the corresponding additional two‐way interaction that did not include time was statistically significant, we left both in the final model. However, if neither the three‐way interaction nor the additional two‐way interaction was significant, we excluded them. To maintain simplicity, three‐way interactions that did not include time were not tested.

We assessed the significance of the full terms with *F*‐tests using the Satterthwaite approximation for the effective degrees of freedom. We performed post‐hoc analyses to assess the effect of each categorical variable and their interactions, where *p*‐values were adjusted for false discovery rate (Benjamini & Hochberg, 1995). Notably, due to the way that variance is partitioned in mixed models (Rights & Sterba, 2019), there is no agreed‐upon approach to calculating standardized effect sizes for individual model terms. Hence, we report unstandardized effect sizes, which align with general recommendations (Pek & Flora, 2018).

## RESULTS

### Missing data analysis

Grade, gender and competence belief of the participants were independent of the membership in either the excluded or the included group for each emotion. For example, when comparing participants who were excluded due to missing data on the happiness variable with those who were not excluded, the chi‐squared test results were as follows: *χ* ^2^(3) = 1.27, *p* = .735 (grade); *χ* ^2^(1) = .01, *p* = .943 (gender); and *χ* ^2^(4) = 5.33, *p* = .255 (competence belief). However, the test score was significantly lower in the excluded participant group than in the included participant group for each emotion. The analysis of variance results were as follows: *t*(2,028) = 7.75, *p* < .001 (happiness); *t*(2,028) = 7.35, *p* < .001 (relaxation); *t*(2,028) = 7.69, *p* < .001 (anxiety) and *t*(2,028) = 7.34, *p* < .001 (boredom). These results suggest that the samples included in the analysis may not fully represent the original sample. It is important to note this limitation when interpreting the results of the mixed‐effects models below.

### Emotion: Happy

**FIGURE 1:**
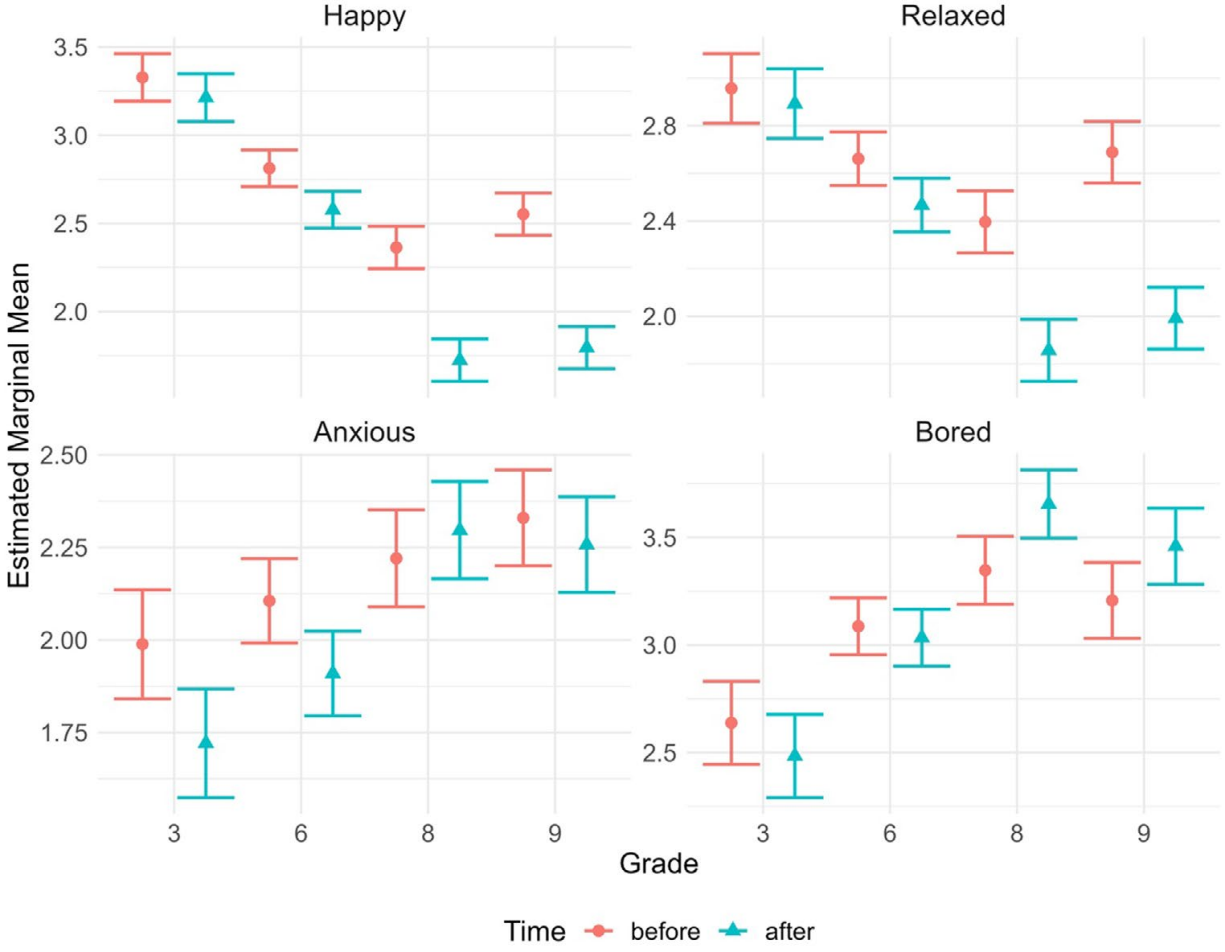
Statistically significant interactions of time and grade level on the levels of the different emotions (happy, relaxed, anxious, bored).

For happiness, the final model included only the main effects of the predictors and the interactions between time and the other predictors (see Table A1). First, the interaction between time and grade (*F*(3; 1,803) = 19.87, *p* < .001) was statistically significant, and post‐hoc analysis revealed that, compared to pre‐assessments, happiness level was significantly lower post‐assessments in grade levels 6, 8 and 9, but not in grade level 3 (see Table 2). The interaction between time and grade on happiness levels is visualized in Figure 1. While the main effects of time (*F*(1; 1,803) = 206.34, *p* < .001) and grade (*F*(3; 1,803) = 80.46, *p* < .001) were significant, they were not meaningful because of the interaction between time and grade as the patterns with respect to time or grade were different depending on which grade level or time point was being examined, respectively.

**TABLE 2:** Results of pairwise comparisons (*t* tests) in students' happiness levels between before and after the assessments, when accounting for gender, grade level and competence belief.

	*N*	Before		After		*t*(1,803)	*p*
		*M*	SD	*M*	SD		
Gender							
Boy	913	3.08	1.21	2.74	1.45	9.06	<.001
Girl	900	2.87	1.21	2.41	1.36	12.63	<.001
Grade level							
3	555	3.69	1.12	3.49	1.34	1.66	.105
6	495	2.95	1.09	2.72	1.29	4.42	<.001
8	355	2.39	1.10	1.77	1.07	10.39	<.001
9	408	2.55	1.10	1.85	1.11	12.37	<.001
Competence belief (‘I am good at mathematics’)							
0	134	2.09	1.28	1.58	1.11	3.63	<.001
1	231	2.40	1.05	1.77	1.07	6.69	<.001
2	386	2.66	1.08	2.23	1.23	7.21	<.001
3	629	3.11	1.12	2.73	1.37	9.84	<.001
4	433	3.64	1.12	3.39	1.38	7.15	<.001
Total	1813	2.98	1.22	2.58	1.42	14.36	<.001

*Note*: For competence belief, 0 corresponds to ‘completely disagree’ and 4 corresponds to ‘completely agree’.

Abbreviations: *M*, mean; *N*, sample size; SD, standard deviation.

**FIGURE 2:**
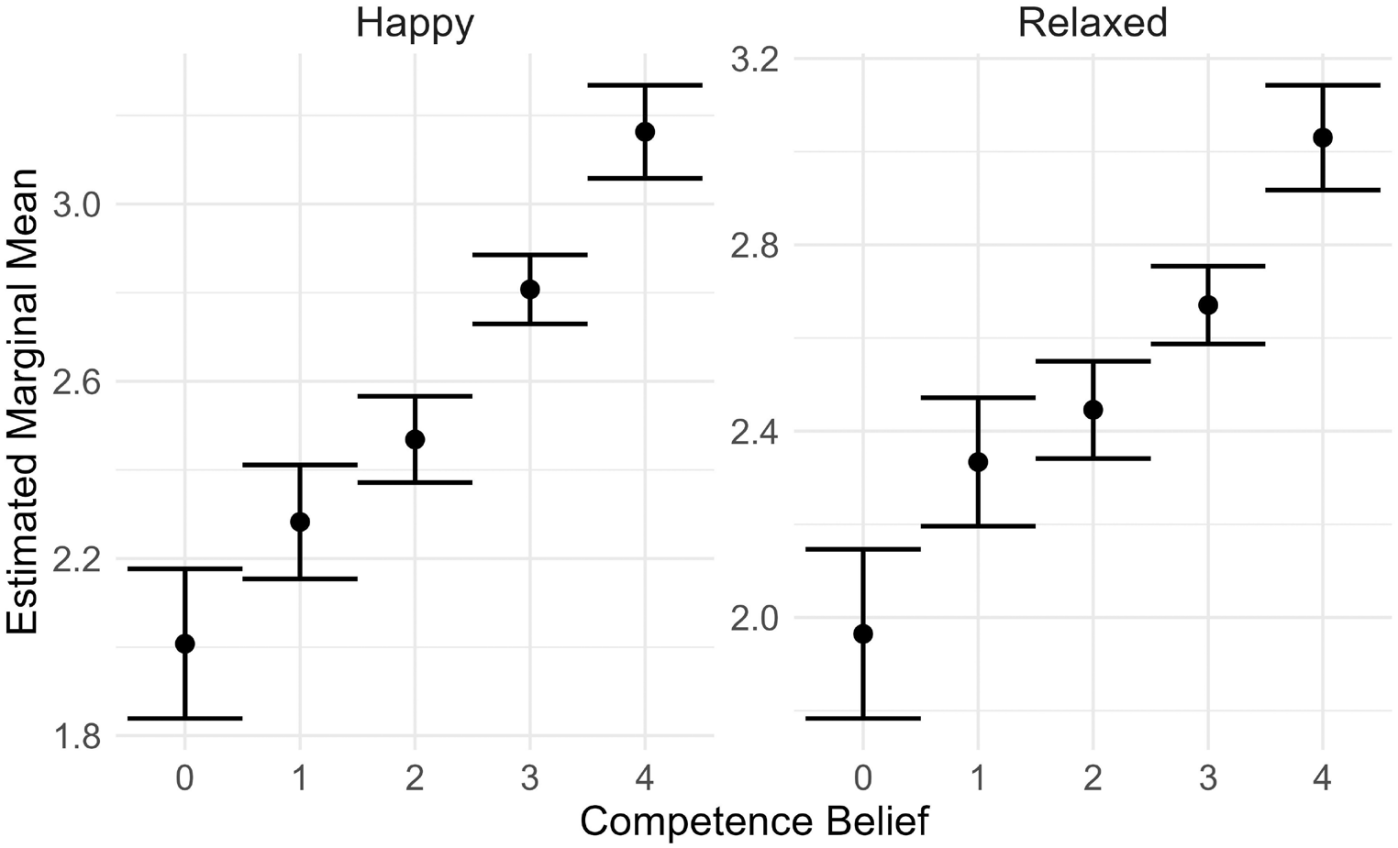
Effect of competence belief (‘I am good at mathematics’; 0 = ‘completely disagree’, 4 = ‘completely agree’) on the levels of positive emotions (happy, relaxed), independent of time.

The interaction between time and gender (*F*(1; 1,803) = 5.02, *p* = .025) was also significant; while happiness level decreased pre‐to‐post assessments for both boys and girls, girls had a larger decrease (see Table 1). The significant main effect of gender (*F*(1; 1,803) = 11.47, *p* = .001) was not meaningful; before the assessments, the difference between boys and girls was not significant (*t*(2,891) = 1.81, *p* = .071), but after the assessments, girls reported lower levels of happiness (*t*(2,891) = 4.05, *p* < .001). Additionally, the interaction between time and competence belief (*F*(4; 1,803) = .44, *p* = .781) was non‐significant, unlike the main effect of competence belief (*F*(4; 1,803) = 40.25, *p* < .001), which is visualized in Figure 2; the level of happiness increased as the level of agreement to being good at mathematics increased, regardless of when the level of happiness was reported.

**FIGURE 3:**
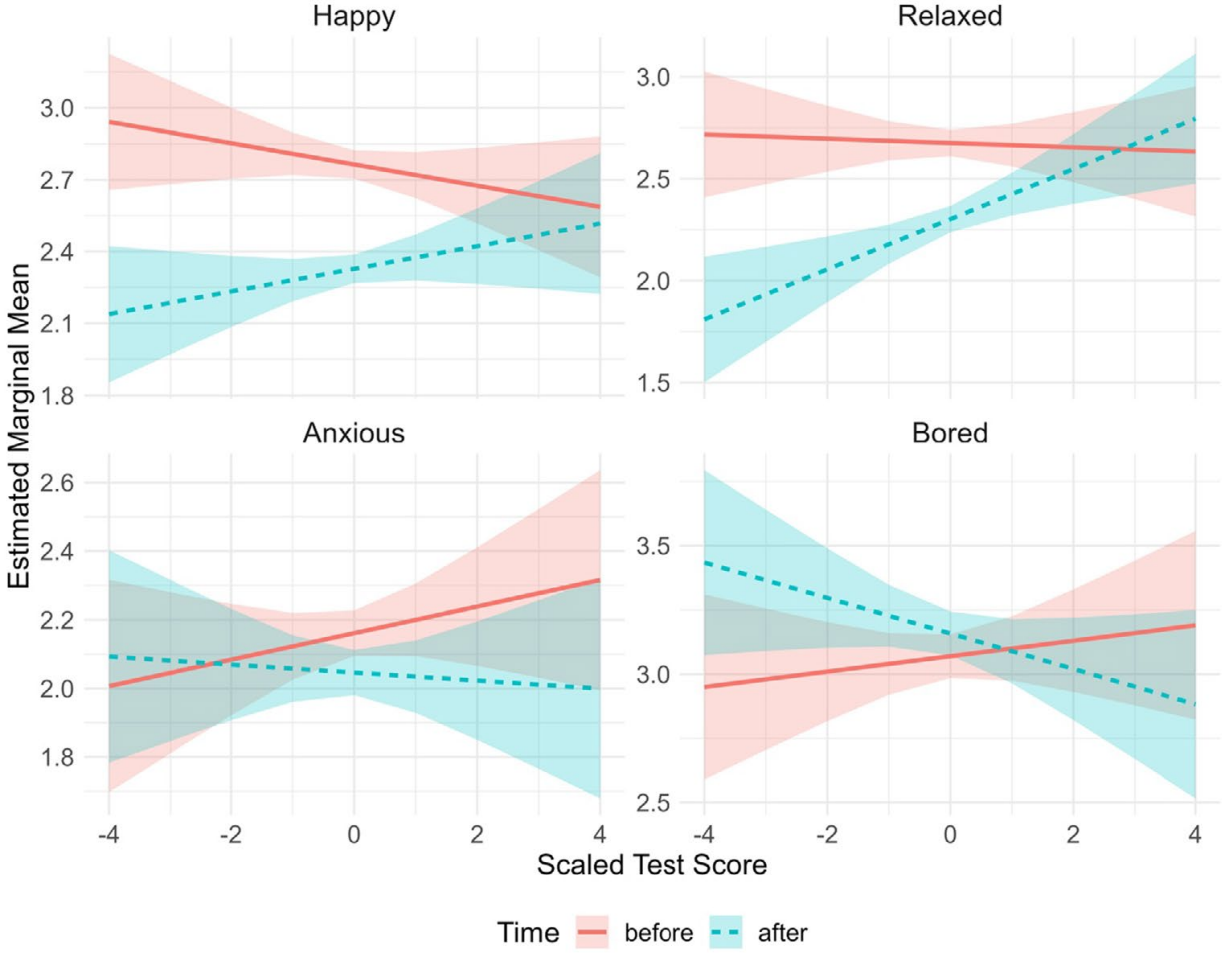
Interaction of time and test score on the levels of the different emotions (happy, relaxed, anxious, bored). Note that the interaction of time and test score on students' anxiety levels was not significant.

Finally, the interaction between time and test score (*F*(1; 1,803) = 6.41, *p* = .011) was significant, but the main effect of test score (*F*(1; 1,803) = .002, *p* = .962) was not: before the assessments, the slope for happiness as a function of test score was negative, whereas post‐assessments, the slope was positive. As seen in Figure 3, low‐performing students (e.g., scaled test score <−2) were happier before than after the assessments, whereas high‐performing students (e.g., scaled test score >2) did not report lower or higher levels of happiness post‐assessments compared to pre‐assessments.

### Emotion: Relaxed

For relaxation, in addition to the main effects and interactions between time and the other predictors, the final model (see Table A2) included a non‐significant interaction term between time, grade and gender (*F*(3; 1,796) = 2.49, *p* = .058). First, similarly to happiness, the interaction between time and grade (*F*(3; 1,796) = 14.29, *p* < .001) was statistically significant (see Figure 1). Post‐hoc analysis indicated that the level of relaxation was significantly lower after the assessments in grade levels 6, 8 and 9, but not in grade level 3 (see Table 3). The non‐significant interaction between time, grade and gender suggested that this relationship between time and grade was gender independent. In addition, like with happiness, the significant main effects of time (*F*(1; 1,796) = 121.23, *p* < .001) and grade (*F*(3; 1,796) = 27.72, *p* < .001) were not meaningful because of the interaction.

**TABLE 3:** Results of pairwise comparisons (*t* tests) in students' relaxation levels between before and after the assessments when accounting for gender, grade level and competence belief.

	*N*	Before		After		*t*(1,796)	*p*
		*M*	SD	*M*	SD		
Gender							
Boy	911	3.06	1.23	2.65	1.40	8.65	<.001
Girl	898	2.64	1.23	2.31	1.30	7.80	<.001
Grade level							
3	557	3.27	1.22	3.03	1.40	.83	.430
6	492	2.80	1.23	2.59	1.33	3.28	.002
8	353	2.43	1.17	1.91	1.15	7.85	<.001
9	407	2.72	1.22	2.10	1.21	10.23	<.001
Competence belief (‘I am good at mathematics’)							
0	132	1.95	1.14	1.67	1.14	1.35	.190
1	230	2.46	1.11	1.91	1.11	5.18	<.001
2	385	2.59	1.21	2.25	1.25	4.91	<.001
3	628	2.94	1.17	2.57	1.34	8.89	<.001
4	434	3.44	1.21	3.10	1.39	7.32	<.001
Total	1809	2.86	1.25	2.48	1.37	11.01	<.001

*Note*: For competence belief, 0 corresponds to ‘completely disagree’ and 4 corresponds to ‘completely agree’.

Abbreviations: *M*, mean; *N*, sample size; SD, standard deviation.

The interaction between time and gender (*F*(1; 1,796) = 1.02, *p* = .313) was non‐significant, unlike the interaction between grade and gender (*F*(3; 1,796) = 4.90, *p* = .002). Girls reported lower levels of relaxation in grades 6, 8 and 9, but there was no such difference in grade level 3 (see Table 4). Since the three‐way interaction between time, grade and gender was non‐significant, these differences between boys and girls applied at a general level independent of time. The significant main effect of gender (*F*(1; 1,796) = 34.63, *p* < .001) was meaningless due to the interaction with grade.

**TABLE 4:** Results of pairwise comparisons (*t* tests) in students' relaxation levels averaged over time between boys and girls when accounting for grade level.

Grade level	Boys			Girls			*t*(1,796)	*p*
	*N*	*M*	SD	*N*	*M*	SD		
3	550	3.22	1.31	564	3.08	1.33	.23	.849
6	510	2.99	1.32	474	2.38	1.17	5.16	<.001
8	344	2.41	1.25	362	1.94	1.07	3.60	.001
9	418	2.58	1.31	396	2.22	1.17	2.72	.010
Total	1822	2.86	1.34	1796	2.47	1.28	5.89	<.001

Abbreviations: *M*, mean; *N*, sample size; SD, standard deviation.

The interaction between time and competence belief in mathematics (*F*(4; 1,796) = 2.30, *p* = .057) was not significant, but the main effect of competence belief (*F*(4; 1,796) = 25.75, *p* < .001) was. The level of relaxation increased as the level of agreement to being good at mathematics increased, regardless of time (see Figure 2). Finally, the main effect of test score (*F*(1; 1,796) = 2.83, *p* = .093) was non‐significant, unlike the interaction between time and test score (*F*(1; 1,796) = 10.91, *p* < .001); the slope for relaxation as a function of test score was negative pre‐assessments, whereas the slope was positive post‐assessments (see Figure 3). Low‐performing students were more relaxed before than after the assessments, whereas high‐performing students did not report differing levels of relaxation between pre‐ and post‐assessments.

### Emotion: Anxious

For anxiety, in addition to the main effects and interactions between time and the other predictors, the final model (see Table A3) included non‐significant interaction terms between time, gender and test score (*F*(1; 1,785) = .20, *p* = .653) and time, gender and competence belief (*F*(4; 1,785) = 1.43, *p* = .222). The interactions between time and grade (*F*(3; 1,785) = 4.51, *p* = .004) (see Figure 1) and time and gender (*F*(1; 1,785) = 5.74, *p* = .017) were significant. Post‐hoc analyses showed significantly reduced anxiety levels in 3rd and 6th graders post‐assessments, whereas levels in 8th and 9th graders remained unchanged (see Table 5). The main effects of time (*F*(1; 1,785) = 11.83, *p* = .001) and grade (*F*(3; 1,785) = 9.59, *p* < .001) were significant but meaningless due to their interaction. Girls' anxiety levels decreased significantly from pre‐ to post‐assessments, whereas boys' anxiety levels remained the same (see Table 5). However, regardless of time, girls reported higher levels of anxiety (before: *t*(2,840) = −7.28, *p* < .001; after: *t*(2,840) = −4.90, *p* < .001). The non‐significant three‐way interactions between time, gender and competence belief and time, gender and test score suggested that the interaction between time and gender was independent of competence belief and test score.

**TABLE 5:** Results of pairwise comparisons (*t* tests) in students' anxiety levels between before and after the assessments when accounting for gender, grade level and competence belief.

	*N*	Before		After		*t*(1,785)	*p*
		*M*	SD	*M*	SD		
Gender							
Boy	905	1.87	1.14	1.77	1.20	.69	.490
Girl	895	2.31	1.26	2.10	1.31	4.48	<.001
Grade level							
3	551	1.85	1.14	1.59	1.05	3.59	.001
6	491	2.05	1.18	1.84	1.17	3.40	.002
8	350	2.23	1.29	2.28	1.42	−1.14	.308
9	408	2.34	1.26	2.23	1.37	1.10	.315
Competence belief (‘I am good at mathematics’)							
0	133	2.66	1.51	2.51	1.60	1.58	.165
1	229	2.28	1.25	2.29	1.40	−.18	.941
2	384	2.18	1.17	2.06	1.30	1.90	.092
3	622	2.02	1.16	1.85	1.16	2.79	.014
4	432	1.83	1.16	1.58	1.07	2.36	.034
Total	1800	2.09	1.22	1.94	1.27	3.44	.001

*Note*: For competence belief, 0 corresponds to ‘completely disagree’ and 4 corresponds to ‘completely agree’.

Abbreviations: *M*, mean; *N*, sample size; SD, standard deviation.

**FIGURE 4:**
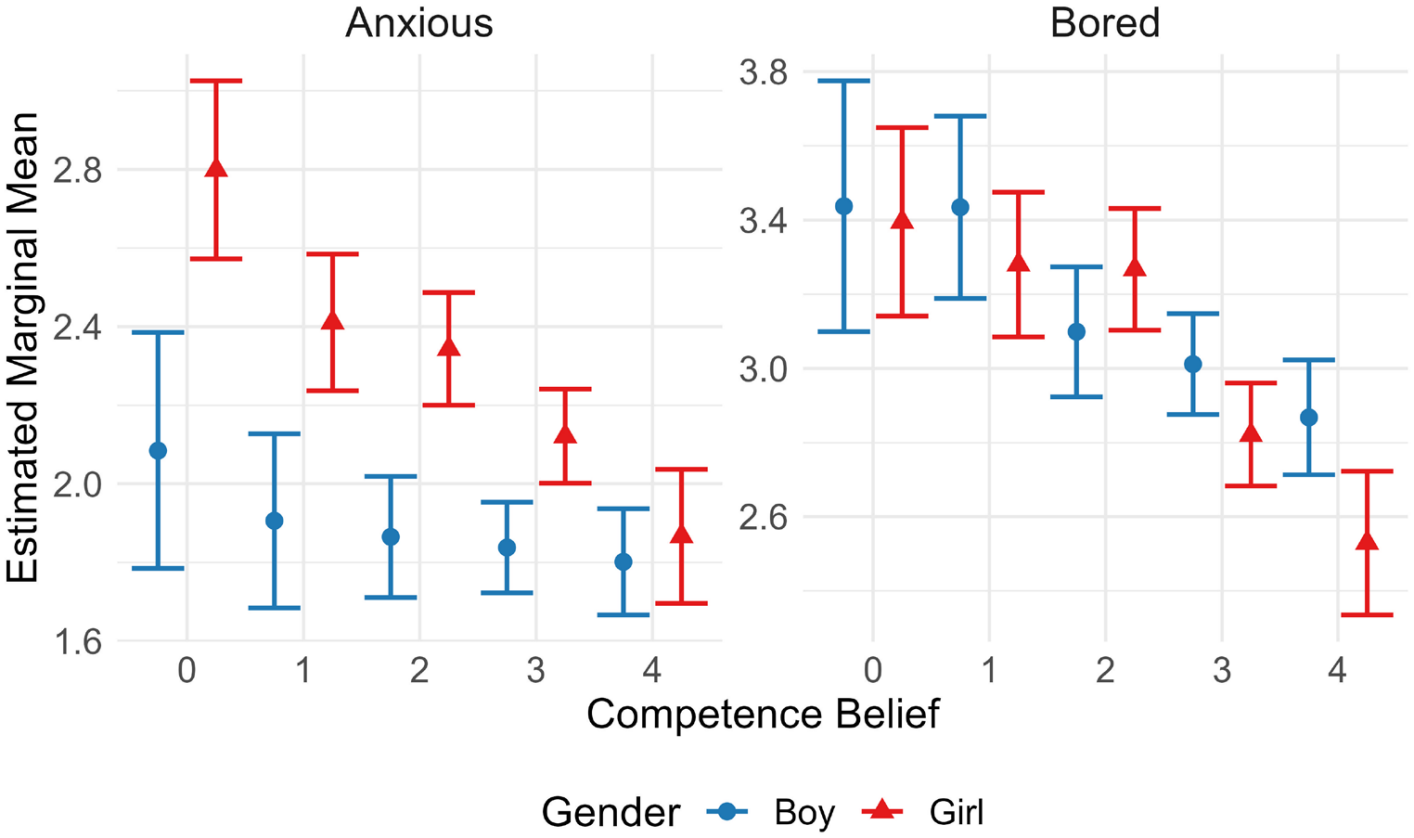
Interaction of gender and competence belief (‘I am good at mathematics’; 0 = ‘completely disagree’, 4 = ‘completely agree’) on the levels of the negative emotions (anxious, bored), independent of time.

**FIGURE 5:**
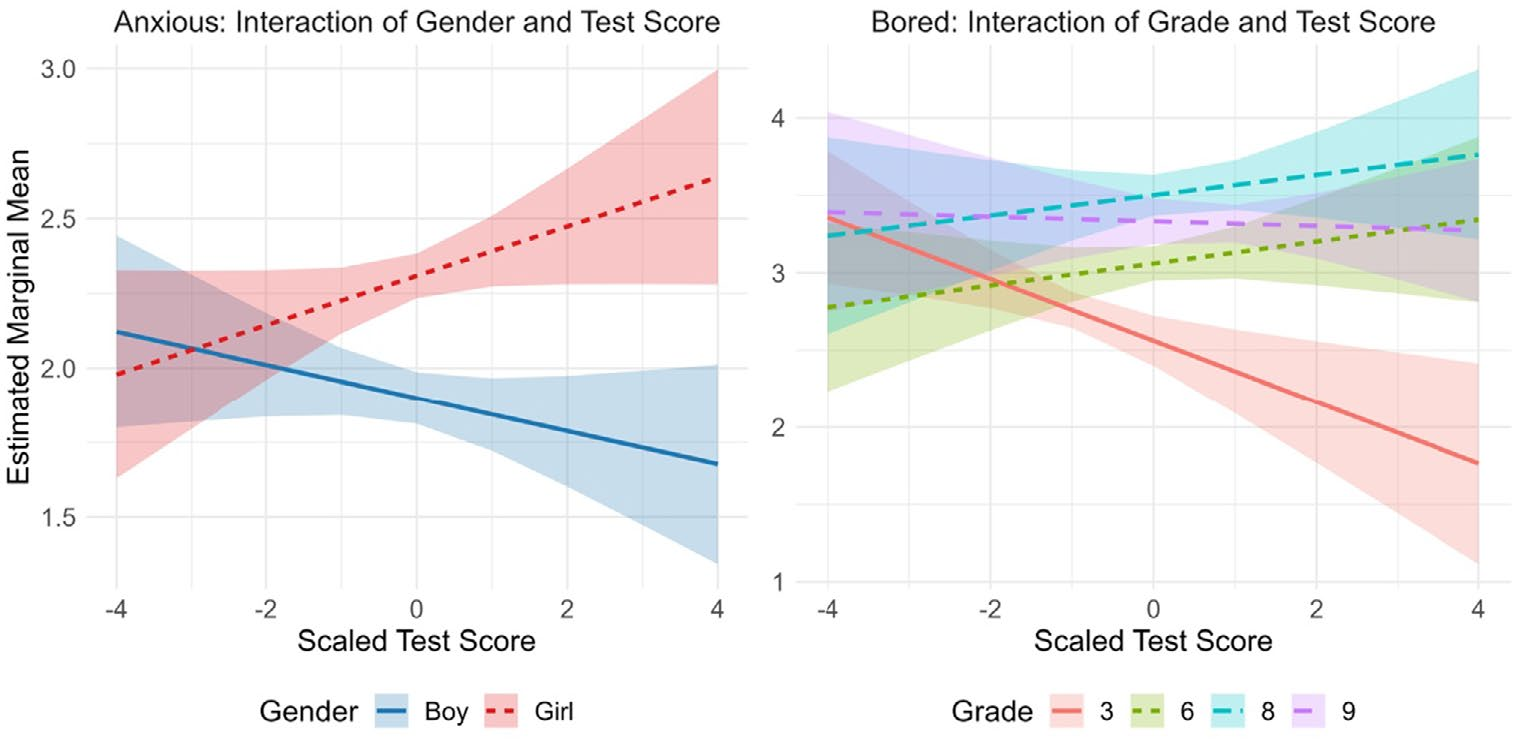
Interaction of gender and test score on the students' general level of anxiety, and interaction of grade and test score on the students' general level of boredom, both of which were independent of time.

The interactions between gender and competence belief (*F*(4; 1,785) = 3.54, *p* = .007) and gender and test score (*F*(1; 1,785) = 7.63, *p* = .006) were significant, and because of the non‐significance of the three‐way interactions including time in addition to these variables, these two‐way interactions were independent of time (see Figures 4 and 5, respectively). Based on post‐hoc analysis, the difference between boys' and girls' anxiety levels averaged over time appeared to decrease as the level of agreement to being good at mathematics increased (see Table 6); among students who perceived low math competence, girls reported higher levels of anxiety, whereas among students with high perceived math competence, girls and boys reported similar levels of anxiety. Due to this interaction, the significant main effects of gender (*F*(1; 1,785) = 49.23, *p* < .001) and competence belief (*F*(4; 1,785) = 7.26, *p* < .001) were not meaningful. Finally, among girls, the slope of anxiety level as a function of test score was positive, whereas among boys, the slope was negative; high‐performing girls were more anxious than high‐performing boys, whereas low‐performing boys and girls reported similar levels of anxiety, regardless of time. The main effect of test score (*F*(1; 1,785) = .16, *p* = .693) was not significant.

**TABLE 6:** Results of pairwise comparisons (*t* tests) in students' anxiety levels averaged over time between boys and girls when accounting for their competence beliefs (‘I am good at mathematics’).

Competence belief	Boys			Girls			*t*(1,785)	*p*
	*N*	*M*	SD	*N*	*M*	SD		
0	94	2.18	1.56	172	2.81	1.51	−3.77	.001
1	176	2.00	1.26	282	2.46	1.34	−3.57	.001
2	356	1.90	1.12	412	2.31	1.30	−4.48	<.001
3	642	1.79	1.13	602	2.08	1.18	−3.39	.002
4	542	1.68	1.12	322	1.76	1.13	−.63	.675
Total	1810	1.82	1.17	1790	2.20	1.29	−7.02	<.001

*Note*: For competence belief, 0 corresponds to ‘completely disagree’ and 4 corresponds to ‘completely agree’.

Abbreviation*s*: *M*, mean; *N*, sample size; SD, standard deviation.

### Emotion: Bored

For boredom, in addition to the main effects and interactions between time and the other predictors, the final model (see Table A4) included non‐significant interaction terms between time, grade and test score (*F*(3; 1,786) = .92, *p* = .431) and time, gender and competence belief (*F*(4; 1,786) = 2.36, *p* = .051). The interactions between time and grade (*F*(3; 1,786) = 5.56, *p* < .001) (Figure 1), time and test score (*F*(1; 1,786) = 4.06, *p* = .044) and grade and test score (*F*(3; 1,786) = 3.58, *p* = .013) were all statistically significant, while the three‐way interaction including these three variables was not, suggesting that each two‐way interaction was independent of the third variable. Based on post‐hoc analysis, boredom levels of 8th and 9th graders increased significantly from pre‐ to post‐assessments, whereas boredom levels of 3rd and 6th graders remained at the same level (see Table 7). The main effect of time (*F*(1; 1,786) = 3.48, *p* = .062) was not significant, and while the main effect of grade (*F*(3; 1,786) = 27.26, *p* < .001) was significant, it was not meaningful in the presence of the interaction.

**TABLE 7:** Results of pairwise comparisons (*t* tests) in students' boredom levels between before and after the assessments when accounting for gender, grade level and competence belief.

	*N*	Before		After		*t*(1,786)	*p*
		*M*	SD	*M*	SD		
Gender							
Boy	907	3.01	1.38	3.07	1.58	−.71	.573
Girl	896	2.92	1.33	3.06	1.49	−2.17	.092
Grade level							
3	556	2.51	1.36	2.51	1.48	1.43	.177
6	491	3.00	1.33	2.98	1.43	.72	.470
8	350	3.36	1.27	3.64	1.47	−3.50	.001
9	406	3.20	1.29	3.43	1.51	−2.57	.015
Competence belief (‘I am good at mathematics’)							
0	130	3.52	1.48	3.57	1.60	.81	.486
1	230	3.41	1.28	3.55	1.48	−1.12	.331
2	384	3.12	1.27	3.37	1.45	−2.74	.011
3	626	2.84	1.30	2.96	1.48	−2.19	.045
4	433	2.60	1.40	2.55	1.52	−.71	.539
Total	1803	2.96	1.36	3.07	1.53	−1.86	.063

*Note*: For competence belief, 0 corresponds to ‘completely disagree’ and 4 corresponds to ‘completely agree’.

Abbreviations: *M*, mean; *N*, sample size; SD, standard deviation.

The interaction between time and test score (see Figure 3) reveals an inverse pattern compared to happiness and relaxation; pre‐assessments, the slope for boredom as a function of test score was positive, whereas post‐assessments, the slope was negative. In other words, low‐performing students were more bored after the assessments, whereas high‐performing students reported similar levels of boredom before and after. Moreover, the interaction between grade and test score (see Figure 5) captures a negative slope for boredom level as a function of test score for the 3rd graders, and positive or near‐zero for others. That is, for the 3rd graders, a higher test score was associated with a lower level of boredom in general, no matter when the level of boredom was reported. The main effect of test score (*F*(1; 1,786) = .27, *p* = .607) was not significant.

Finally, the interaction between gender and competence belief (*F*(4; 1,786) = 2.56, *p* = .037) was significant and independent of time (see Figure 4). Among students who did not believe to be good at mathematics (‘completely disagree’, ‘disagree’), no significant difference could be found between boys' and girls' levels of boredom (see Table 8). Conversely, girls who believed to be good at mathematics (‘completely agree’) reported significantly lower levels of boredom than boys with similar beliefs. Due to this interaction, the significant main effect of competence belief (*F*(4; 1,786) = 13.03, *p* < .001) was not meaningful. The main effect of gender (*F*(1; 1,786) = 2.94, *p* = .087) was not significant.

**TABLE 8:** Results of pairwise comparisons (*t* tests) in students' boredom levels averaged over time between boys and girls when accounting for their competence beliefs (‘I am good at mathematics’).

Competence belief	Boys			Girls			*t*(1,786)	*p*
	*N*	*M*	SD	*N*	*M*	SD		
0	92	3.58	1.65	168	3.52	1.49	.20	.882
1	178	3.59	1.46	282	3.41	1.33	.99	.417
2	358	3.20	1.39	410	3.29	1.34	−1.42	.221
3	644	3.00	1.43	608	2.79	1.34	2.05	.067
4	542	2.71	1.50	324	2.35	1.35	2.95	.008
Total	1814	3.04	1.48	1792	2.99	1.42	1.71	.087

*Note*: For competence belief, 0 corresponds to ‘completely disagree’ and 4 corresponds to ‘completely agree’.

Abbreviations: *M*, mean; *N*, sample size; SD, standard deviation.

## DISCUSSION

Our results suggest that students' emotions associated with math testing are dynamic and affected by several student characteristics. First, we see significant differences in the emotional states from before to after the assessment. For all students except 3rd graders, levels of positive emotions (happiness, relaxation) generally decreased from pre‐ to post‐assessments, contrasting previous findings and our hypothesis H1 of post‐assessment‐relaxation (Goetz et al., 2007; Pekrun et al., 2002). Anxiety, on the other hand, followed a similar decreasing trend for elementary school students, while middle school students still reported similar levels of anxiety after the test. Conversely, middle school students' boredom levels increased from before to after the assessment, whereas elementary school students' boredom levels remained the same. This partially supports H1, as happiness and anxiety, representing the activating emotions, were at higher level before the test for some groups of the students. Moreover, the results were well aligned with previous findings of decreasing enjoyment (positive activating emotion) and increasing boredom (negative deactivating emotion) from pre‐to‐post‐testing by Goetz et al. (2007).

Nonetheless, particularly among older students, we see a decrease in positive emotions, accompanied by an increase in negative deactivating emotions after the assessment. Younger students expressed plausibly more stable emotional states, however, including a decrease in anxiety after the assessment. This suggests their pre‐assessment anxiety may differ from that of older students—possibly more state‐like, dynamic and sensitive to environmental changes (cf. Halme et al., 2022; Pekrun et al., 2018). Overall, these findings support H2, indicating that individual factors influence emotional shifts.

Second, gender was a significant predictor of emotional states. In general, aligned with previous findings, boys experienced higher levels of positive emotions and lower levels of anxiety (Erturan & Jansen, 2015; Frenzel et al., 2007; Justicia‐Galiano et al., 2023). While there were no gender differences in happiness levels before the assessment, boys were happier than girls afterwards. Boys also experienced higher levels of relaxation, both before and after the assessment, whereas, conversely, girls reported higher levels of anxiety. Aligning with the findings of Erturan and Jansen (2015), we observed that gender differences in test anxiety evened out if girls perceived higher math competence beliefs. This might indicate higher trait‐like test anxiety among girls, previously proposed by Goetz et al. (2013).

Third, aligning with prior research (Haciomeroglu, 2019; Liu et al., 2018; Van Der Beek et al., 2017), students' math competence beliefs indicated differences in the general levels of students' emotions. Students who perceived themselves as more competent in math experienced higher levels of positive emotions. Our findings also align with previous research pointing to a significant decreasing trend in the level of students' mathematics competence belief across the early years of elementary school to secondary level education (Nagy et al., 2010), as younger students broadly experienced more positive emotions.

Additionally, we observed an impact of math competence belief on students' negative emotions in interaction with other student characteristics; high math competence was associated with lower anxiety and boredom, yet girls with low competence beliefs were highly anxious, and girls with high competence beliefs were even less bored than boys with high competence beliefs. These findings support our hypothesis H3, indicating that individual factors impact students' emotional states during assessment in a similar way they predict math‐related emotions overall. The results may point to a hypothesis that particularly girls with low self‐perceived math competence could plausibly benefit from additional affective scaffolding, supporting their regulation of negative emotions, during mathematics learning. However, further research is needed to support this hypothesis. Competence belief was strongly associated with performance, as a post hoc test with a linear regression model with standardized test score as the dependent variable and gender (*F*(1; 2,020) = 2.24, *p* = .135), competence belief (*F*(4; 2,020) = 11.97, *p* < .001) and their interaction (*F*(4; 2,020) = .34, *p* = .850) as predictors, reveals that students who had higher competence beliefs performed better on the test.

Based on our findings, students who performed poorly showed a decline in positive emotions post‐assessment, suggesting that low performance feels emotionally discouraging. The finding also supports our hypotheses H2 and H3, portraying test‐performance as a significant predictor of (changes in) students' emotional states. Additionally, as an indication of heightened anxiety among girls (cf. Frenzel et al., 2007), high‐performing girls experienced more anxiety than high‐performing boys, while lower‐performing girls experienced similar levels of anxiety to low‐performing boys. Conversely, poorly performing students experienced an increase in negative deactivating boredom. We suggest that this is a maladaptive coping strategy, demonstrated by over‐challenged students, which might be linked to the experience of low control over the situation (D'Mello et al., 2014; Kleine et al., 2005; Pekrun et al., 2010; Schwartze et al., 2020). However, for 3rd graders, high‐performing students experienced less boredom in general, indicating that the assessment may have been more engaging for them (cf. Bekker et al.). Perhaps high‐performers among older students may experience greater pressure and stress than their younger peers (Parviainen et al., 2021; Salmela‐Aro et al., 2018), which could foster a more cynical attitude towards learning—a tendency reflected in their higher levels of boredom (Bekker et al., 2023; Ramos‐Vera et al., 2025).

Although the participants of this study did not receive any formal feedback during the test, they may have engaged in self‐evaluation of their performance. Based on previous studies, self‐reflection and self‐assessment can also function as feedback (Vogl & Pekrun, 2016) and influence students' emotional levels (Tulis & Ainley, 2011). In general, success feedback tends to elicit positive emotions, while failure feedback can trigger negative affects (Tulis & Ainley, 2011). Previous research also states that boys tend to react more adaptively to their errors than girls (Soncini et al., 2022). Our findings contrast with this result as we did not observe any gender differences between the shifts from pre‐ to post‐assessment for lower‐performing students. Encouraging feedback provided in a digital learning environment has been found to mitigate students' negative emotional responses to failure (Narciss & Alemdag, 2025; Soncini et al., 2025; Tulis & Dresel, 2025), which could also be utilized in digital assessments. This could support students' adaptive reactions, promoting continuous learning following the assessment. Educators should, however, bear in mind that some negative emotions, experienced at a moderate level, can be linked to higher agency and increase students' performance by activating more efficient metacognitive strategies (Pekrun et al., 2002; Tulis & Fulmer, 2013).

Consequently, instead of evading negative emotions, educators should invest in supporting students' meta‐affective learning (Radoff et al., 2019; Sharabi & Roth, 2025). This means promoting behavioural shifts in students towards seeing frustration and challenges as excellent learning opportunities—transforming the negative affective experiences into positive meta‐affects. As a result, facing challenges or disappointment in one's performance can act as a driver for better performance in the future. Particularly, students who are attentive to negative emotions and demonstrate adaptive coping practices are likely to learn from their academic failure (Sharabi & Roth, 2025). Based on the observed maladaptive reactions to academic failure among low performers, we suggest that they would particularly benefit from interventions supporting meta‐affective learning (cf. Burleson, 2006).

Finally, although much of the previous research has been conducted in low‐stakes testing settings, our findings align with that. This supports a good generalizability of the findings on students' emotions associated with mathematics testing across various settings and environments.

### Limitations and future research

A limitation is that this was the first pilot of the digital assessment, making the setting unfamiliar for many students, likely affecting their experiences (cf. Putwain, 2008). The length and content of the test may have impacted students' emotional levels, but generally, both were designed according to what students are generally used to at each grade level. Furthermore, there are limitations related to the generalizability of the results. Originally, 3387 students from 12 municipalities representing three of the six geographical areas and two out of three types of municipalities participated. Thus, the dataset accurately represents Southern and Southwestern Finland and Lapland, but the results cannot be generalized to the eastern, western, inland and northern areas of Finland. Overall, the results can also be generalized to urban municipalities and municipalities with high population density, but not to rural municipalities. Moreover, the final sample included in the analysis may not have fully represented the original sample. This is because we found that those who were excluded due to missing data in the emotion variables achieved statistically significantly lower test scores than those who were not excluded.

Additionally, students' background math competence beliefs were measured at the same time as their before‐assessment‐emotions. Accordingly, these before‐assessment‐emotions could have impacted students' competence beliefs. This sets limitations on interpreting the directions of the interactions, and in future studies, students' background information should be measured before testing. This could provide clearer perspectives on, for example, the role of internalized, more general math competence in predicting students' pre‐assessment‐emotions. Furthermore, students' competence beliefs and emotions were measured with one item, and therefore, measurement errors could not be modelled (see Gogol et al., 2014). Nevertheless, based on previous research, for measuring motivational‐affective constructs, such as emotions competence belief, single‐item indicators offer a psychometrically sound alternative to longer scales (see Allen et al., 2022; Goetz et al., 2016; Gogol et al., 2014; Hoeppner et al., 2011). For example, the single‐item, ‘I am good at mathematics’, used in this study expresses good predictive validity of students' mathematics self‐concept based on previous studies (cf. Metsämuuronen, 2012; Fennema & Sherman, 1976).

Additionally, state and trait‐like anxiety might operate differently and have a different impact on students' performance (cf. Halme et al., 2022; Tulis & Ainley, 2011). In this study, we were not able to identify the stability of the emotion and explicitly distinguish, for example, trait‐like test or math anxiety from state anxiety. This should be considered in future studies focusing on the dynamics of emotional trajectories. Finally, the emotion items appeared several times during the test, which could have affected the emotional landscapes of students. However, we only measured the levels of emotions before and after the assessment tasks. Future studies should consider the emotional trajectories during testing, providing a deeper understanding of students' emotional responses to specific tasks.

## CONCLUSIONS

This study examined how students' levels of four emotions (i.e., happiness, relaxation, anxiety and boredom) develop from before to after assessment, investigating the role of individual factors (i.e., gender, grade level, perceived mathematical competence and test performance) in predicting students' emotional states and moderating their emotional trajectories. The sample (*N* = 2179) consisted of students from various schools across Finland, who participated in a digital, semi‐high‐stakes, end‐of‐year mathematics assessment. Students' emotions were measured immediately before and after the assessment using an in‐situ approach, and all analyses were conducted using linear mixed‐effects modelling. The results point out that students reported lower positive emotions after the assessment. However, the measured individual factors significantly predicted both students' emotional states and how they developed during the assessment. Younger students remained more positive during the assessment and even experienced a decrease in anxiety after. In general, boys reported higher levels of positive emotions and lower anxiety and students who perceived themselves as competent experienced higher levels of positive and lower levels of negative emotions. Finally, students who performed poorly showed a decline in positive emotions during the assessment. We suggest that students with heightened negative emotions would benefit from additional emotional support, fostering meta‐affective learning, after completing a higher‐stakes assessment.

## AUTHOR CONTRIBUTIONS

**Reetta Kyynäräinen:** Conceptualization; writing – original draft; writing – review and editing; visualization. **Santeri Holopainen:** Formal analysis; methodology; visualization; writing – original draft; writing – review and editing. **Jari Metsämuuronen:** Conceptualization; investigation; project administration; writing – review and editing. **Umar Bin Qushem:** Conceptualization; writing – review and editing. **Mikko‐Jussi Laakso:** Funding acquisition; project administration. **Katarina Alanko:** Conceptualization; investigation; project administration; writing – review and editing.

## CONFLICT OF INTEREST STATEMENT

No potential conflict of interest was reported by the authors.

## Data Availability

The data that support the findings of this study are available on request from the corresponding author. The data are not publicly available due to privacy or ethical restrictions.

## Full results of the final mixed‐effects models

**TABLE A1:** Full results of the final mixed‐effects model for happiness.

Fixed effect	Beta	SE	95% CI	*t*(1,803)	*p*
Intercept (grand mean)	2.55	.03	2.49 to 2.60	97.18	<.001
Time (before)	.22	.02	.19 to .25	14.36	<.001
Grade (3)	.72	.05	.62 to .83	13.83	<.001
Grade (6)	.15	.04	.07 to .22	3.9	<.001
Grade (8)	−.5	.04	−.59 to −.41	−11.21	<.001
Gender (boy)	.08	.02	.03 to .12	3.39	.001
CB (0)	−.54	.07	−.68 to −.40	−7.43	<.001
CB (1)	−.26	.06	−.38 to −.15	−4.57	<.001
CB (2)	−.08	.05	−.17 to .01	−1.67	.096
CB (3)	.26	.04	.18 to .34	6.34	<.001
Test score	0	.03	−.06 to .06	.05	.962
Time (before) × Grade (3)	−.16	.03	−.22 to −.10	−5.3	<.001
Time (before) × Grade (6)	−.1	.02	−.14 to −.06	−4.53	<.001
Time (before) × Grade (8)	.1	.03	.05 to .15	3.9	<.001
Time (before) × Gender (boy)	−.03	.01	−.06 to −.00	−2.24	.025
Time (before) × CB (0)	−.04	.04	−.12 to .05	−.88	.379
Time (before) × CB (1)	.04	.03	−.03 to .10	1.07	.284
Time (before) × CB (2)	−.01	.03	−.06 to .04	−.4	.688
Time (before) × CB (3)	.01	.02	−.04 to .05	.33	.745
Time (before) × Test score	−.05	.02	.08 to −.01	−2.53	.011

*Note*: Test score is scaled (*M* = 0, SD = 1). Variance (SD) of the random intercept for participants was .61 (.78). Marginal and conditional *R*‐squared values were .31 and .65, respectively. *p*‐Values for fixed effects were calculated using Satterthwaite approximations. Model equation: Happiness level ~ (1 | Participant) + Time + Grade + Gender + Competence belief + Test score + Time × (Grade + Gender + Competence belief + Test score).

Abbreviation: CB, competence belief.

**TABLE A2:** Full results of the final mixed‐effects model for relaxation.

Fixed effect	Beta	SE	95% CI	*t*(1,796)	*p*
Intercept (grand mean)	2.49	.03	2.43 to 2.54	88.52	<.001
Time (before)	.19	.02	.15 to .22	11.01	<.001
Grade (3)	.44	.06	.33 to .55	7.77	<.001
Grade (6)	.08	.04	−.01 to .16	1.83	.067
Grade (8)	−.36	.05	−.46 to −.27	−7.56	<.001
Gender (boy)	.15	.02	.10 to .20	5.89	<.001
CB (0)	−.52	.08	−.68 to −.37	−6.73	<.001
CB (1)	−.16	.06	−.28 to −.03	−2.52	.012
CB (2)	−.04	.05	−.14 to .05	−.87	.386
CB (3)	.18	.04	.09 to .27	4.11	<.001
Test score	.06	.03	−.01 to .12	1.68	.093
Time (before) × Grade (3)	−.16	.03	−.22 to −.09	−4.58	<.001
Time (before) × Grade (6)	−.09	.02	−.14 to −.04	−3.61	<.001
Time (before) × Grade (8)	.08	.03	.03 to .14	2.86	.004
Time (before) × Gender (boy)	.02	.02	−.01 to .04	1.01	.313
Grade (3) × Gender (boy)	−.14	.04	−.21 to −.06	−3.47	.001
Grade (6) × Gender (boy)	.09	.04	.01 to .17	2.29	.022
Grade (8) × Gender (boy)	.05	.05	−.04 to .14	1.11	.268
Time (before) × CB (0)	−.11	.05	−.20 to −.02	−2.37	.018
Time (before) × CB (1)	.03	.04	−.04 to .11	.87	.383
Time (before) × CB (2)	−.03	.03	−.09 to .03	−.97	.334
Time (before) × CB (3)	.04	.03	−.01 to .09	1.54	.124
Time (before) × Test score	−.07	.02	−.11 to −.03	−3.3	.001
Time (before) × Grade (3) × Gender (boy)	.01	.02	−.04 to .05	.29	.77
Time (before) × Grade (6) × Gender (boy)	−.05	.02	−.09 to .00	−1.82	.069
Time (before) × Grade (8) × Gender (boy)	−.03	.03	−.08 to .03	−.92	.36

*Note*: Test score is scaled (*M* = 0, SD = 1). Variance (SD) of the random intercept for participants was .67 (.82). Marginal and conditional *R*‐squared values were .18 and .56, respectively. *p*‐Values for fixed effects were calculated using Satterthwaite approximations. Model equation: Relaxation level ~ (1 | Participant) + Time + Grade + Gender + Competence belief + Test score + Grade × Gender + Time × (Grade + Gender + Competence belief + Test score + Grade × Gender).

Abbreviation: CB, competence belief.

**TABLE A3:** Full results of the final mixed‐effects model for anxiety.

Fixed effects	Beta	SE	95% CI	*t*(1,785)	*p*
Intercept (grand mean)	2.1	.03	2.05 to 2.16	72	<.001
Time (before)	.06	.02	.02 to .09	3.44	.001
Gender (boy)	−.2	.03	−.26 to −.15	−7.02	<.001
Test score	.01	.03	−.05 to .08	.39	.693
CB (0)	.34	.08	.18 to .50	4.16	<.001
CB (1)	.05	.06	−.07 to .18	.86	.392
CB (2)	0	.05	−.10 to .10	.01	.991
CB (3)	−.12	.05	−.21 to −.04	−2.74	.006
Grade (3)	−.25	.06	−.36 to −.14	−4.37	<.001
Grade (6)	−.1	.04	−.18 to −.01	−2.31	.021
Grade (8)	.15	.05	.06 to .25	3.18	.002
Time (before) × Gender (boy)	−.04	.02	−.07 to −.01	−2.4	.017
Time (before) × Test score	.03	.02	−.01 to .06	1.29	.197
Gender (boy) × Test score	−.07	.02	−.12 to −.02	−2.76	.006
Time (before) × CB (0)	.03	.05	−.06 to .12	.65	.514
Time (before) × CB (1)	−.06	.04	−.14 to .01	−1.77	.076
Time (before) × CB (2)	0	.03	−.06 to .06	.04	.967
Time (before) × CB (3)	.01	.03	−.04 to .06	.43	.668
Gender (boy) × CB (0)	−.15	.08	−.31 to .00	−1.93	.054
Gender (boy) × CB (1)	−.05	.06	−.17 to .07	−.77	.439
Gender (boy) × CB (2)	−.03	.05	−.13 to .06	−.69	.491
Gender (boy) × CB (3)	.06	.04	−.02 to .15	1.44	.151
Time (before) × Grade (3)	.08	.03	.01 to .14	2.34	.02
Time (before) × Grade (6)	.04	.02	−.01 to .09	1.7	.089
Time (before) × Grade (8)	−.1	.03	−.15 to −.04	−3.42	.001
Time (before) × Gender (boy) × Test score	−.01	.01	−.03 to .02	−.45	.653
Time (before) × Gender (boy) × CB (0)	−.01	.05	−.10 to .08	−.19	.847
Time (before) × Gender (boy) × CB (1)	−.07	.04	−.14 to .00	−1.86	.064
Time (before) × Gender (boy) × CB (2)	.03	.03	−.02 to .09	1.21	.228
Time (before) × Gender (boy) × CB (3)	0	.02	−.05 to .05	−.09	.925

*Note*: Test score is scaled (*M* = 0, SD = 1). Variance (SD) of the random intercept for participants was .73 (.85). Marginal and conditional *R*‐squared values were .09 and .55, respectively. *p*‐values for fixed effects were calculated using Satterthwaite approximations. Model equation: Anxiety level ~ (1 | Participant) + Time + Grade + Gender + Competence belief + Test score + Gender × (Test score + Competence belief) + Time × [Grade + Gender + Competence belief + Test score + Gender × (Test score + Competence belief)].

Abbreviation: CB, competence belief.

**TABLE A4:** Full results of the final mixed‐effects model for boredom.

Fixed effects	Beta	SE	95% CI	*t*(1,786)	*p*
Intercept (grand mean)	3.11	.04	3.04 to 3.19	85.43	<.001
Time (before)	−.04	.02	−.09 to .00	−1.86	.062
Grade (3)	−.55	.07	−.69 to −.42	−8.07	<.001
Grade (6)	−.05	.05	−.15 to .04	−1.07	.285
Grade (8)	.39	.06	.27 to .50	6.6	<.001
Test score	−.02	.04	−.09 to .05	−.51	.607
Gender (boy)	.06	.03	−.01 to .12	1.71	.087
CB (0)	.3	.09	.12 to .48	3.3	.001
CB (1)	.24	.07	.10 to .38	3.4	.001
CB (2)	.07	.06	−.04 to .18	1.21	.225
CB (3)	−.2	.05	−.30 to −.10	−3.91	<.001
Time (before) × Grade (3)	.12	.04	.03 to .21	2.72	.007
Time (before) × Grade (6)	.07	.03	.01 to .13	2.16	.031
Time (before) × Grade (8)	−.11	.04	−.18 to −.04	−2.88	.004
Time (before) × Test score	.05	.02	.00 to .10	2.01	.044
Grade (3) × Test score	−.18	.06	−.29 to −.07	−3.12	.002
Grade (6) × Test score	.09	.06	−.02 to .20	1.6	.11
Grade (8) × Test score	.09	.06	−.03 to .20	1.39	.163
Time (before) × Gender (boy)	.02	.02	−.02 to .06	.96	.337
Time (before) × CB (0)	.1	.06	−.02 to .22	1.71	.088
Time (before) × CB (1)	−.02	.05	−.11 to .08	−.33	.742
Time (before) × CB (2)	−.07	.04	−.14 to .00	−1.84	.065
Time (before) × CB (3)	−.03	.03	−.10 to .03	−.93	.351
Gender (boy) × CB (0)	−.03	.09	−.21 to .14	−.39	.695
Gender (boy) × CB (1)	.02	.07	−.11 to .16	.31	.755
Gender (boy) × CB (2)	−.14	.06	−.25 to −.03	−2.49	.013
Gender (boy) × CB (3)	.04	.05	−.06 to .13	.81	.416
Time (before) × Grade (3) × Test score	.03	.04	−.04 to .11	.87	.385
Time (before) × Grade (6) × Test score	−.05	.04	−.12 to .02	−1.38	.169
Time (before) × Grade (8) × Test score	.04	.04	−.04 to .11	.89	.372
Time (before) × Gender (boy) × CB (0)	.08	.06	−.03 to .19	1.41	.158
Time (before) × Gender (boy) × CB (1)	−.13	.04	−.22 to −.05	−2.97	.003
Time (before) × Gender (boy) × CB (2)	.04	.04	−.03 to .11	1.15	.252
Time (before) × Gender (boy) × CB (3)	.01	.03	−.05 to .07	.3	.766

*Note*: Test score is scaled (*M* = 0, SD = 1). Variance (SD) of the random intercept for participants was .77 (.88). Marginal and conditional R‐squared values were .11 and .47, respectively. *p*‐Values for fixed effects were calculated using Satterthwaite approximations. Model equation: Boredom level ~ (1 | Participant) + Time + Grade + Gender + Competence belief + Test score + Grade × Test score + Gender × Competence belief + Time × (Grade + Gender + Competence belief + Test score + Grade × Test score + Gender × Competence belief).

Abbreviation: CB, competence belief.
